# Silicate-substituted strontium apatite nano coating improves osteogenesis around artificial ligament

**DOI:** 10.1186/s12891-019-2777-8

**Published:** 2019-08-31

**Authors:** Takuya Egawa, Yusuke Inagaki, Manabu Akahane, Akira Furukawa, Kazuya Inoue, Munehiro Ogawa, Yasuhito Tanaka

**Affiliations:** 1 Department of Orthopedic Surgery, Nara Medical University, Shijocho 840, Kashihara, Nara, 634-8522 Japan; 2 Department of Artificial Joint and Regenerative Medicine for Bone and Cartilage, Nara Medical University, Shijocho 840, Kashihara, Nara, 634-8522 Japan; 3 Department of Public Health, Health Management and Policy, Nara Medical University, Shijocho 840, Kashihara, Nara, 634-8522 Japan

**Keywords:** Silicate-substituted strontium, Anterior cruciate ligament injuries, Nano coating, Osteogenesis, PET artificial ligament

## Abstract

**Background:**

Treatment of anterior cruciate ligament injuries commonly involves the use of polyethylene terephthalate (PET) artificial ligaments for reconstruction. However, the currently available methods require long fixation periods, thereby necessitating the development of alternative methods to accelerate the healing process between tendons and bones. Thus, we developed and evaluated a novel technique that utilizes silicate-substituted strontium (SrSiP).

**Methods:**

PET films, nano-coated with SrSiP, were prepared. Bone marrow mesenchymal cells (BMSCs) from femurs of male rats were cultured and seeded at a density of 1.0 × 10^4^/cm^2^ onto the SrSiP-coated and non-coated PET film, and subsequently placed in an osteogenic medium. The osteocalcin concentration secreted into the medium was compared in each case. Next, PET artificial ligament, nano-coated with SrSiP, were prepared. BMSCs were seeded at a density of 4.5 × 10^5^/cm^2^ onto the SrSiP-coated, and non-coated artificial ligament, and then placed in osteogenic medium. The osteocalcin and calcium concentrations in the culture medium were measured on the 8th, 10th, 12th, and 14th day of culture. Furthermore, mRNA expression of osteocalcin, alkaline phosphatase (ALP), bone morphogenetic protein-2 (BMP2), and runt-related transcription factor 2 (Runx2) was evaluated by qPCR. We transplanted the SrSiP-coated and non-coated artificial ligament to the tibiae of mature New Zealand white rabbits. Two months later, we sacrificed them and histologically evaluated them.

**Results:**

The secretory osteocalcin concentration in the medium on the film was significantly higher for the SrSiP group than for the non-coated group. Secretory osteocalcin concentration in the medium on the artificial ligament was also significantly higher in the SrSiP group than in the non-coated group on the 14th day. Calcium concentration on the artificial ligament was significantly lower in the SrSiP group than in the non-coated group on the 8th, 10th, 12th, and 14th day. In qPCR as well, OC, ALP, BMP2, and Runx2 mRNA expression were significantly higher in the SrSiP group than in the non-coated group. Newly formed bone was histologically found around the artificial ligament in the SrSiP group.

**Conclusions:**

Our findings demonstrate that artificial ligaments using SrSiP display high osteogenic potential and thus may be efficiently used in future clinical applications.

## Background

Anterior cruciate ligament (ACL) injuries are the most frequent sports trauma in the field of orthopedic surgery and sports medicine. The “anatomical double-bundle ACL reconstruction,” which utilizes the knee flexor tendon, is one of the standard techniques, in which autogenic tendon is directly connected to a polyethylene terephthalate (PET) artificial ligament passing through the bone tunnels of the tibia [1, 2]. Of course, in ACL reconstruction, the sole use of autologous tendon is the first choice. However, in the anatomical double-bundle ACL reconstruction, the artificial ligaments are required to fashion the two auto-grafts because of the relatively lower amount of harvested tendons than that with single bundle ACL reconstruction. Although this method is surgically less invasive and provides excellent intraoperative maneuverability, the long time period required for the fixation between the transplanted tendon and bone tunnel remains a challenge. Alternative treatment options, to speed up the healing process, have been studied, for example, using periosteum, calcium phosphate, hyperbaric oxygen and growth factors, or mesenchymal stem cells (MSCs) [3–10]. In previous reports, hydroxyapatite (HAP) was coated on the surface of the artificial ligament using various methods, and studies were conducted with the purpose of imparting biocompatibility to the surface and improving osseointegration [11–13]. However, there has been no report till date regarding the use of apatite with introduced strontium (Sr) on the artificial ligament. PET artificial ligament can be coated with HAP using our novel nano coating methods. HAP is chemically represented as Ca_10_ (PO_4_) _6_ (OH) _2_; Calcium (Ca) may be substituted with Sr, and phosphate ion (PO_4_^2−^) with silicate ions (SiO_4_^4−^) to enhance osteogenic ability.

The null hypothesis of this study was that there is no difference between the SrSiP-coated and the non-coated artificial ligaments in terms of bone formation ability.

## Methods

### Preparation of the PET film and the PET artificial ligament with SrSiP coating

Strontium hydrogen phosphate (SrHPO_4_) was prepared as a precursor of the following synthesis of SrSiP from a mixture of equimolar solutions of strontium chloride and diammonium hydrogen phosphate ((NH_4_)_2_HPO_4_). The obtained SrHPO_4_ was dispersed in water, a half molar amount of sodium metasilicate (Na_3_SiO_3_) was added to it, and the temperature of the mixture was raised to 90 °C. The reaction mixture was subjected to vigorous stirring for 5 h and then left to stand at ambient temperature. The precipitated product was filtered and washed several times with 1 N sodium hydroxide solution and finally with distilled water. The product was dried at 70 °C overnight. The chemical composition and crystal structure of the product were determined by XRD and ICP-AES, respectively, and the product was confirmed to be silicate substituted strontium apatite. A rotation/revolution mixer (Nano Pulverizer NP-100, Thinky Co., Ltd., Tokyo, Japan) was used to prepare nano-sized apatite dispersion in acetonitrile. One gram of apatite was introduced into the vessel inside the mixer and 0.2 g of poly (DL-lactic acid) (BMG Incorporated, Kyoto, Japan), dissolved in 10 mL of acetonitrile and 10 mL of zirconia (Y_2_O_3_-ZrO_2_) balls with diameter of 0.3 mm (YTZ-0.3) (Nikkato Co., Ltd. Osaka, Japan), were added. Wet milling was performed at 2000 rpm for a few minutes, and the resultant dispersion was filtered from zirconia balls. The PET film was dipped in the above apatite solution (concentration adjusted to 5 wt%) and withdrawn thereafter. Excess solution was removed, and the coated sample was left to dry in an oven at 70 °C for 3 h [14]. After drying, the film was cut into pieces 12 mm in diameter.

The PET artificial ligament was dipped in the above apatite solution (solution concentration was adjusted to 5 wt%) and withdrawn thereafter. Excess solution was removed and the coated sample was left to dry in an oven at 70 °C for 3 h.

### Experiment 1: culture experiment

#### Experiment using the PET film

##### Cell culture for seeding on the PET film

All animal handling and surgical procedures were conducted according to the Nara Medical University Institutional Animal Care and Use Committee Statement. Cell culture was performed as reported previously [15–18]. Bone marrow was harvested from both femurs of 7-week-old Fisher-344 male rats (Japan SLC, Shizuoka, Japan). Rats were sacrificed using 4% isoflurane (Pfizer, Tokyo, Japan), which they inhaled for 5 min while inside a sealed container; in addition, 50 mg/kg pentobarbital (Kyoritsu Seiyaku, Tokyo, Japan) was injected into their peritoneal cavities. The deaths of the animals were confirmed by cardiac arrest, respiratory arrest, and loss of corneal reflex. An initial culture was started in a flask. After 2 weeks, cells that attached to the culture dish and proliferated were collected as bone marrow mesenchymal cells (BMSCs) using trypsin-ethylenediaminetetraacetic acid (EDTA) solution (0.25% trypsin, 0.53 mM EDTA-4Na; Nacalai Tesque, Kyoto, Japan). Minimal Essential Medium containing 15% fetal bovine serum and antibiotic was used as a standard culture medium. The osteogenic culture medium consisted of the above standard culture medium, 10 nM dexamethasone, 0.28 mM ascorbic acid phosphate, and 10 mM β-glycerophosphate. BMSCs were seeded at a density of 1.0 × 10^4^/cm^2^ onto the SrSiP nano-coated PET film (and non-coated PET film as control), placed in a 24-well plate (Falcon, BD Biosciences, Franklin Lanes, NJ, USA) in the osteogenic medium. Culture continued for 14 days. Cultures were maintained in a humidified atmosphere with 95% air and 5% CO_2_ at 37 °C.

### Secretory osteocalcin concentrations measurement

Osteocalcin concentrations in 14-day culture supernatants were measured by ELISA method (rat osteocalcin ELISA kit DS, DS Pharma Biomedical Co., Osaka, Japan) [15] (*n* = 10).

#### Experiment using the PET artificial ligament

##### Observation of artificial ligament

The artificial ligament was observed immediately after coating, using low vacuum- subsequent scanning electron microscopy (SEM) (SU3500, Hitachi, Japan) equipped with an energy dispersive X-ray spectrometer (EDS) (Octane Plus, Ametek Inc. U.S.A.), with acceleration voltage of 20 kV at 60 Pa. The artificial ligament was also observed at 50× magnification using SEM. Elemental analysis of the artificial ligament surface was performed using EDS.

##### Cell culture for seeding on the PET artificial ligament

BMSCs were collected and seeded at a density of 4.5 × 10^5^/cm^2^ on the SrSiP nano-coated artificial ligament and non-coated artificial ligament (as control), placed in a 24-well plate in the osteogenic medium. After 14 days, we observed the artificial ligaments in a similar manner to that described above.

##### Secretory osteocalcin and calcium concentration measurement

Secretory osteocalcin concentrations in 8-day, 10-day, 12-day, and 14-day culture supernatants were measured using the ELISA method [15] (*n* = 5).

Calcium concentrations in 8-, 10-, 12-, and 14-day culture supernatants were measured by methyl xylenol blue absorbance method kit (Calcium E-test; Wako Co., Osaka, Japan) according to the manufacturer’s instruction. Reduced calcium in the medium would reflect the amount of calcium deposited. Therefore, calcium concentration in the culture medium could be employed as a marker of osteogenesis in tissue-engineered bone [19].

##### Quantitative polymerase chain reaction (qPCR) in nano-coated PET fiber artificial ligament culture medium

mRNA was extracted from the cells after a culture period of 14 days, and the expression level of osteocalcin, alkaline phosphatase (ALP), Bone Morphogenetic Protein-2 (BMP2), and Runt-related transcription factor 2 (Runx2) was evaluated by qPCR. The expression levels of each target gene were standardized with respect to glyceraldehyde-3-phosphate dehydrogenase (GAPDH) mRNA expression level [16] (*n* = 5).

### Experiment 2: transplantation experiment

#### Histological examination

Two skeletally mature male New Zealand white rabbits, weighting 4150 ± 250 g, were purchased from Japan SLC (Shizuoka, Japan). They were anesthetized by intramuscular injections of 100 mg ketamine (Daiichi Sankyo, Tokyo, Japan) and 20 mg xylazine (Bayer, Osaka, Japan); then, as they inhaled 2% isoflurane, artificial ligament transplantation surgery was performed. Briefly, knee joints were accessed via a lateral parapatellar approach. A 3.2-mm diameter tunnel was created in the proximal tibial metaphysis. The size of the artificial ligaments was 3 × 1 cm. We rolled them and transplanted the SrSiP artificial ligament onto the right knee and the non-coated artificial ligament onto the left knee. Two months later, the rabbits were sacrificed. To sacrifice rabbits, 100 mg ketamine and 20 mg xylazine were injected intramuscularly and then 10 ml potassium chloride saturated solution (Wako Co., Osaka, Japan) was injected intravenously. The deaths of the animals were confirmed by cardiac arrest, respiratory arrest, and loss of corneal reflex. The tibiae for histological evaluation were fixed in 10% neutral buffered formalin, decalcified in EDTA solution, and embedded in paraffin. Sections of 5-μm thickness were prepared in planes parallel to the long axis of the artificial ligament and stained with hematoxylin and eosin.

### Statistical analysis

The results were subjected to statistical analysis via the Mann-Whitney U test using IBM SPSS Statistics 18 (Chicago, Ill., USA). Statistical significance was set at *p* < 0.05. The osteocalcin concentrations in the PET film culture medium and qPCR between the SrSiP and the non-coat groups were compared. Similarly, the osteocalcin and calcium concentrations in the PET artificial ligament culture medium between the SrSiP and the non-coat groups, were compared at each time point.

## Results

### Experiment 1: culture experiment

#### Osteocalcin concentrations in nano-coated PET film culture medium

**Fig. 1:**
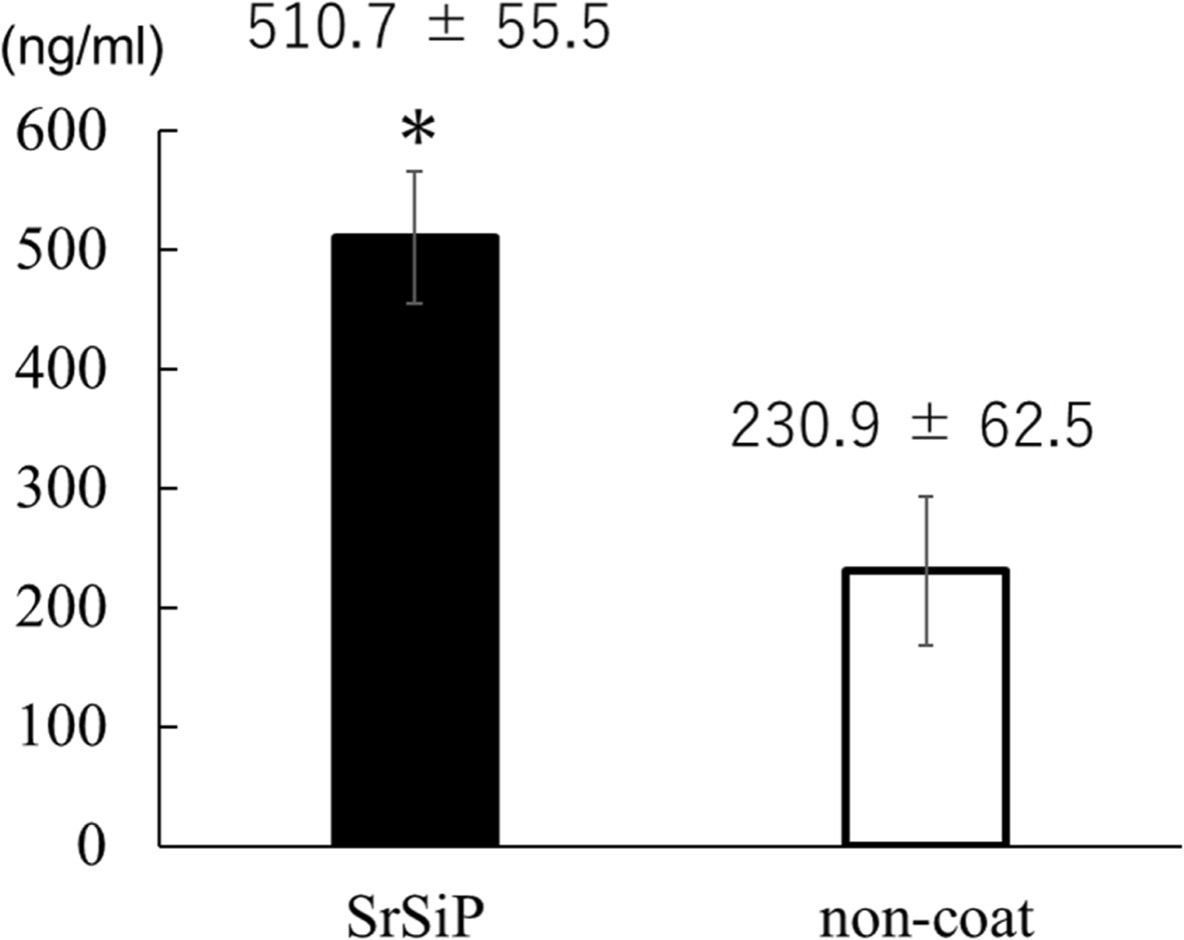
Osteocalcin concentration in nano-coated PET film culture medium. The concentration in SrSiP was significantly higher than in the non-coated sample. Data are shown as the mean ± standard deviation (SD). Asterisk indicates *p* < 0.05

The concentration of osteocalcin in the culture medium was significantly higher in the SrSiP group than in the non-coated group on the 14th day of the culture supernatant (Fig. 1).

#### SEM/EDS analysis of coated artificial ligaments

**Fig. 2:**
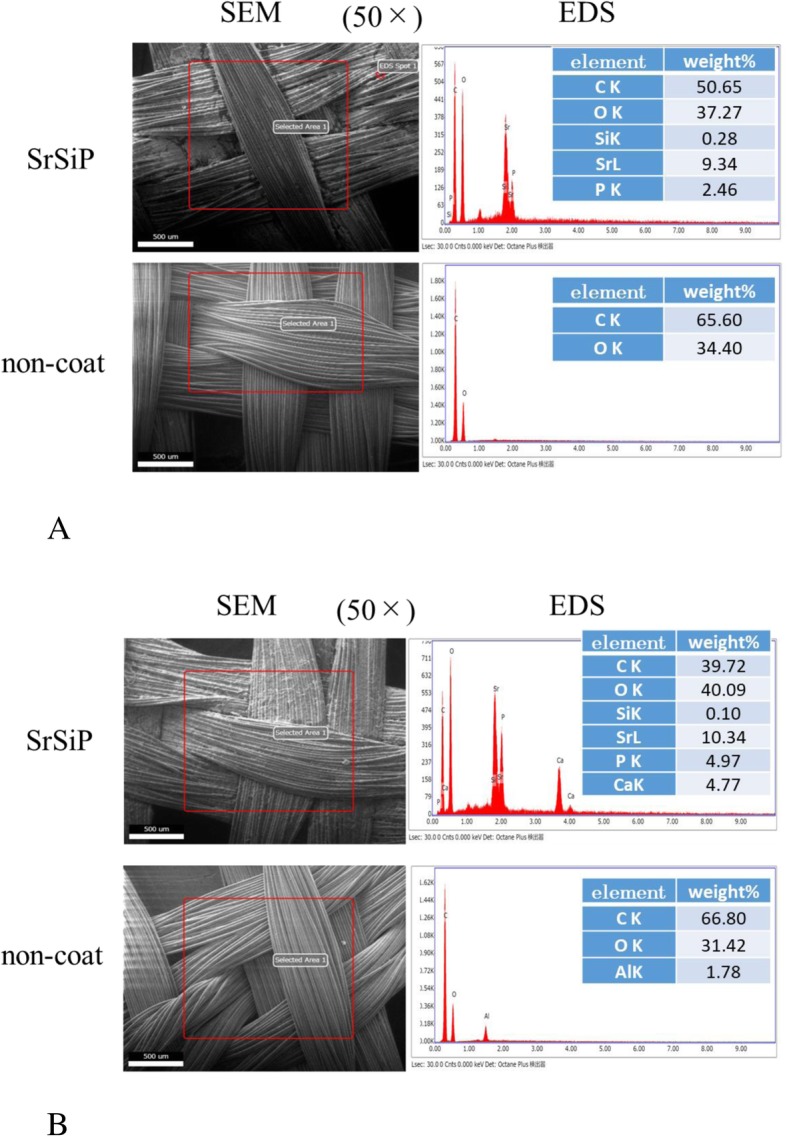
SEM image and EDS results. **a** Before cell culture. **b** After cell culture

SEM, as shown in Fig. 2a upper left, revealed the coating surface of the ligament to be even, and elemental analysis using EDS, as in Fig. 2a upper right showed the presence of surface coating by SrSiP. After cell culture, deposition of calcium phosphate on the SrSiP coat surface was evident by SEM/EDS observations, as shown in Fig. 2b upper right, whereas virtually no calcium deposition was observed in case of the non-coated sample (control) (Fig. 2b right down figure), suggesting increased osteogenesis on the SrSiP surface.

#### Osteocalcin concentrations in nano-coated PET fiber artificial ligament culture medium

**Fig. 3:**
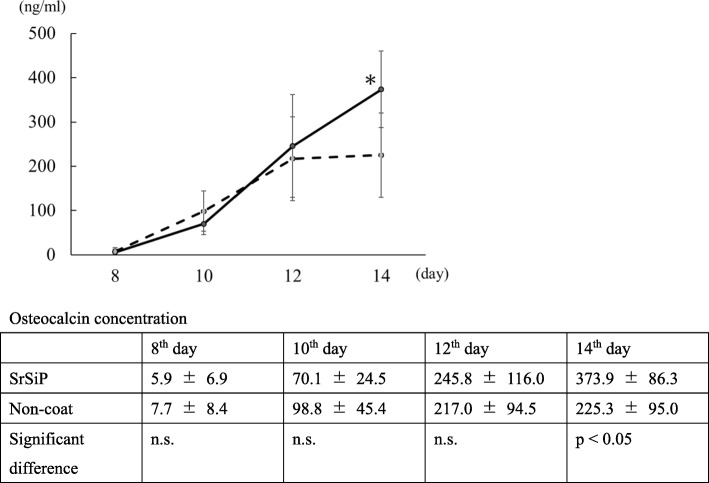
Osteocalcin concentration in nano-coated PET fiber artificial ligament culture medium. Osteocalcin concentration on the 8th, 10th, 12th, and 14th day was recorded. Osteocalcin concentration in SrSiP was significantly higher than in the non-coated sample on the 14th day in the culture supernatant. The solid and broken lines indicate the data obtained in SrSiP and non-coated samples. Data are shown as the mean ± SD. Asterisk indicates p < 0.05 vs. non-coated group (control)

Osteocalcin concentration in the culture medium was significantly higher in the SrSiP group than in the non-coated group on the 14th day of culture supernatant (Fig. 3).

#### Calcium concentrations in nano-coated PET fiber artificial ligament culture medium

**Fig. 4:**
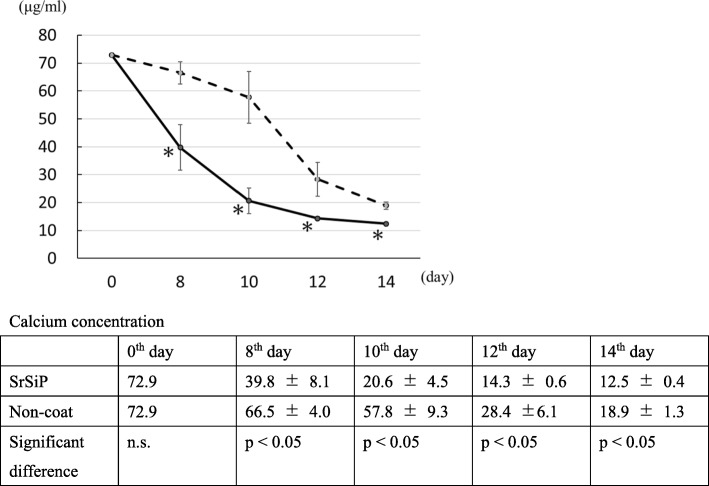
Calcium concentration in nano-coated PET fiber artificial ligament culture medium. Calcium concentration on the 8th, 10th, 12th, and 14th day was recorded. Calcium concentration in SrSiP was significantly lower than in the non-coated sample on the 8th, 10th, 12th, and 14th day of the culture supernatant. The solid and broken lines indicate the data obtained in SrSiP and non-coated samples. Data are shown as the mean ± SD. Asterisk indicates *p* < 0.05 vs. non-coated group (control)

The concentration of calcium in the culture medium was significantly lower in the SrSiP group than in the non-coated group on the 8th, 10th, 12th, and 14th day of culture supernatant (Fig. 4). This result indicates enhanced consumption of calcium ions by osteoblast cells cultured on the apatite-coated ligament surface.

#### qPCR in nano-coated PET fiber artificial ligament culture medium

**Fig. 5:**
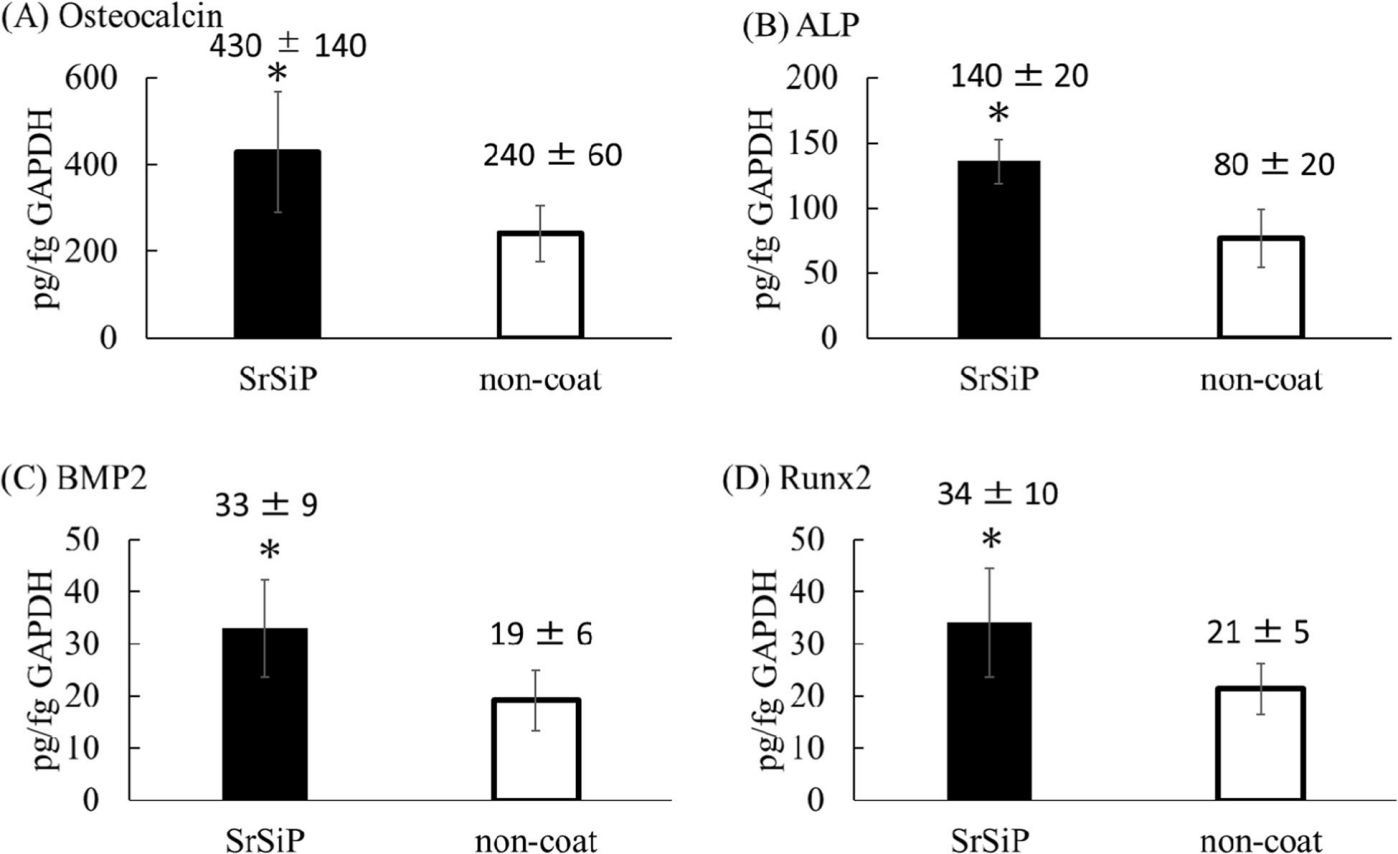
mRNA expression in nano-coated PET fiber artificial ligament. **a** Osteocalcin; **b** ALP; **c** BMP2; **d** Runx2. For Osteocalcin, ALP, BMP2, and Runx2, mRNA expressions in SrSiP were all significantly higher than in the non-coated group. Data are shown as the mean ± SD. Asterisk indicates *p* < 0.05

The mRNA expression level was normalized with respect to that of GAPDH. In qPCR as well, OC, ALP, BMP2, and Runx2 mRNA expression were significantly higher in the SrSiP groups than in the non-coat group (Fig. 5).

### Experiment 2: transplantation experiment

#### Histological findings

**Fig. 6:**
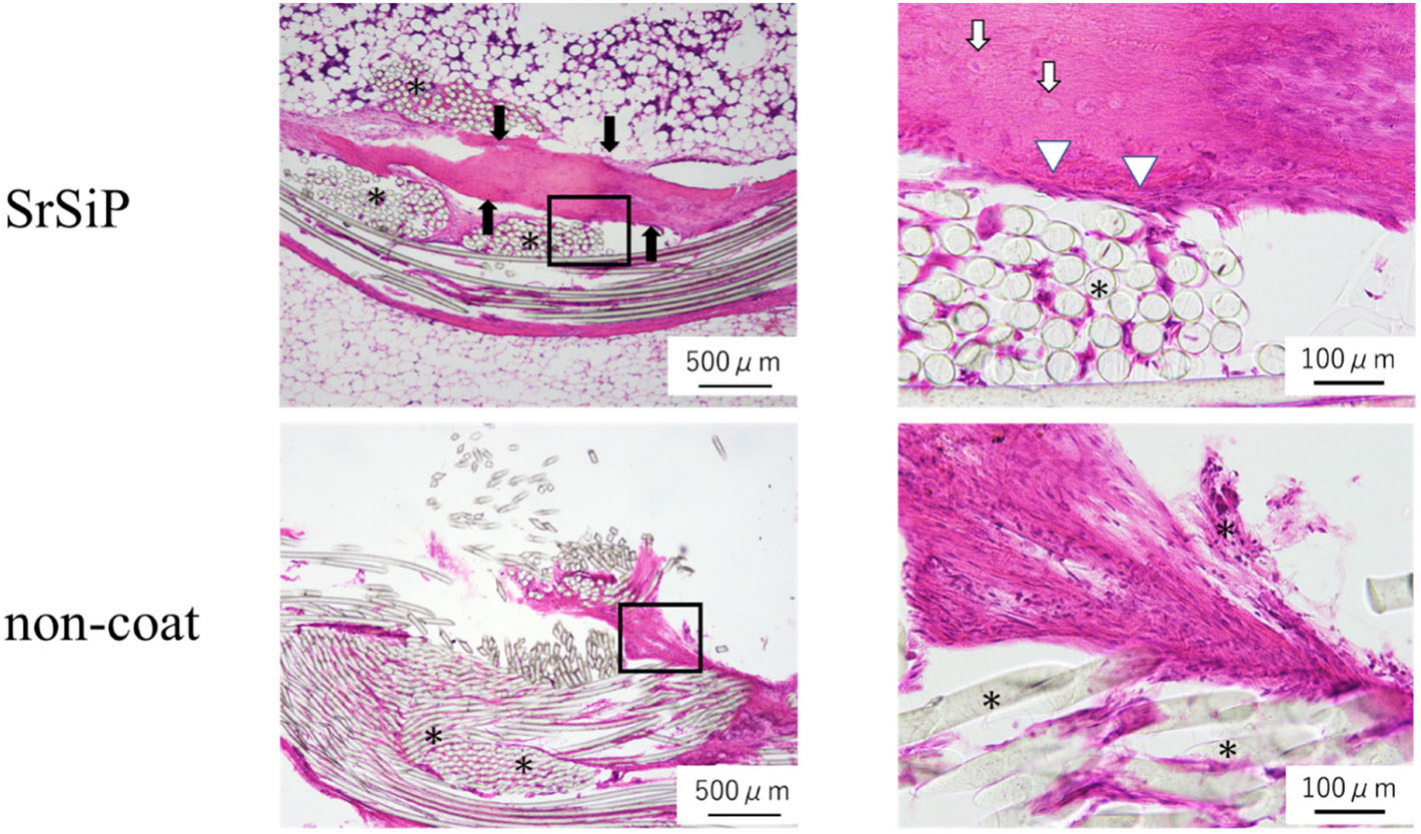
Histological findings. Hematoxylin and eosin stained sections showed newly formed bone around the artificial ligaments of the SrSiP group. The asterisks indicate PET artificial ligament fibers. The black arrows indicate the newly formed bone area. The white arrows indicate osteocytes. The white arrowheads indicate osteoblasts

Newly formed bone was found around the artificial ligaments of SrSiP group. Newly formed bone was stained bright red and purple with hematoxylin and eosin staining. At high magnification, osteoblasts appeared to line up at the edge of bone tissue, in which osteocytes are located. Osteoblasts had abundant basophil cytoplasm. There was an artificial ligament next to it. On the other hand, in the non-coated group, no newly bone was found around the artificial ligaments. Fibroblasts and fibrous tissue were found around the artificial ligaments (Fig. 6).

#### Complications

There was no adverse event such as infection or fracture for all animal experiments.

## Discussion

Sr, an element with an atomic number of 38, belongs to the same family as Ca and stimulates osteogenic differentiation through Ca sensing receptors. Furthermore, by enhancing the secretion of osteoprotegerin, it inhibits the differentiation of osteoclasts. It inhibits bone resorption by preventing the differentiation of preosteoblast into osteoclast via RANKL [20]. Therefore, strontium ranelate has been clinically applied as a dual-action bone agent for the treatment of osteoporosis in Europe [21]. Recently, the widespread use of strontium ranelate has been discontinued in most countries, owing to the concerns regarding the potential cardiovascular risk, although this remains somewhat controversial [22]. However, some studies have demonstrated that strontium-doped medical applications do benefit bone metabolism, leading to improved bone healing and osseointegration with lesser side effects than in systemic administration [23].

Silicon is the second most abundant element on the Earth’s crust [24]. In a study using human osteoblast cells, accumulation of orthosilicic acid in cells was shown to promote the synthesis of collagen type 1 and differentiation into osteoblasts [25]. Furthermore, silicon nanoparticles not only stimulate bone formation in osteoblasts but also have inhibitory effects on osteoclasts [26]. Silicon actively participates in initial bone formation [27], and the addition of Silicon to biomaterials is known to enhance their bioactivity [28] and osteogenic properties [29, 30].

In this study, coating PET film with SrSiP was confirmed to promote osteogenic potential. Uniform surface coating on the artificial ligament by the nano-sized apatite dispersion was also demonstrated. These apatite coatings enhanced calcium consumption, hence promoting subsequent deposition of calcium phosphate on the coated surface of the artificial ligaments. In particular, even coating of the nanoparticulate apatite has been demonstrated to promote the osteogenic potential of BMSCs in the SrSiP group as compared to the non-coated group. The newly formed bone around the artificial ligament was histologically shown in the SrSiP group.

In this study, SrSiP coating promoted the maximum bone formation. It is significant to be able to nano-coat strontium and silicon. Application of SrSiP nano-coating would lead to the development of new biomaterial with high osteogenic potential, thereby boosting the field of orthopedic surgery and sports medicine, with potential clinical applications in ACL reconstruction.

There are several limitations to this study. First, a biomechanical evaluation has not been done. In this study, we tested a new strategy to surface-modify artificial ligament with osteogenic apatite. Basic experiments using cultured cells were conducted mainly to demonstrate the bone formation promoting potential. In the future, in addition to temporal observation in the ligament reconstruction model, we will evaluate this approach with respect to the biomechanical properties and test it in vivo. Second, the concentration of strontium or silicate ion in solution has not been measured. Finally, it is necessary to investigate further whether there is any related adverse event. However, our experimental results suggest the possibility of promotion of early bone formation by SrSiP coating; it would need further validation in a further study.

## Conclusion

In this study, after apatite was synthesized, nanoparticles were formed, existing PET film and artificial ligaments were coated, and the osteogenic potential of the nanoparticles was observed using mesenchymal cells collected from the bone marrow of F 344 rats. Results suggest that SrSiP can promote the osteogenic potential of PET artificial ligament and may be expected to be clinically available in the future as a biomaterial with high osteogenic potential.

## Data Availability

The datasets used during the present study are available from the corresponding author on reasonable request.

## Abbreviations

ACL: Anterior cruciate ligament
ALP: Alkaline phosphatase
BMP2: Bone morphogenetic protein-2
BMSCs: Bone marrow mesenchymal cells
EDS: Energy dispersive X-ray spectrometer
GAPDH: Glyceraldehyde-3-phosphate dehydrogenase
HAP: Hydroxyapatite
PET: Polyethylene terephthalate
qPCR: Quantitative polymerase chain reaction
Runx2: Runt-related transcription factor 2
SD: Standard deviation
SEM: Scanning electron microscop
SrSiP: Silicate-substituted strontium
